# Galectin-3 Inhibits Paracoccidioides brasiliensis Growth and Impacts Paracoccidioidomycosis through Multiple Mechanisms

**DOI:** 10.1128/mSphere.00209-19

**Published:** 2019-04-24

**Authors:** Otavio Hatanaka, Caroline Patini Rezende, Pedro Moreno, Fabrício Freitas Fernandes, Patrícia Kellen Martins Oliveira Brito, Roberto Martinez, Carolina Coelho, Maria Cristina Roque-Barreira, Arturo Casadevall, Fausto Almeida

**Affiliations:** a Department of Biochemistry and Immunology, Ribeirao Preto Medical School, University of Sao Paulo, Ribeirao Preto, SP, Brazil; b Department of Cellular and Molecular Biology, Ribeirao Preto Medical School, University of Sao Paulo, Ribeirao Preto, SP, Brazil; c Department of Internal Medicine, Ribeirao Preto Medical School, University of Sao Paulo, Ribeirao Preto, SP, Brazil; d Department of Biosciences, College of Life and Environmental Sciences, University of Exeter, Exeter, United Kingdom; e Medical Research Council Centre for Medical Mycology, University of Aberdeen, Aberdeen, United Kingdom; f Department of Molecular Microbiology and Immunology, Johns Hopkins Bloomberg School of Public Health, Baltimore, Maryland, USA; Carnegie Mellon University

**Keywords:** Paracoccidioides brasiliensis, extracellular vesicles, fungal infection, galectin-3

## Abstract

The thermodimorphic pathogenic fungi Paracoccidioides brasiliensis and Paracoccidioides lutzii are the etiologic causes of paracoccidioidomycosis (PCM), the most prevalent systemic mycosis in Latin America. Galectin-3 (Gal-3), an animal β-galactoside-binding protein, modulates important roles during microbial infections, such as triggering a Th2-polarized immune response in PCM. Herein, we demonstrate that Gal-3 also plays other important roles in *P. brasiliensis* infection. We verified that Gal-3 levels are upregulated in human and mice infections and established that Gal-3 inhibited *P. brasiliensis* growth by inhibiting budding. Furthermore, Gal-3 affected disruption and internalization of extracellular vesicles (EVs) from *P. brasiliensis* by macrophages. Our results suggest important protective roles for Gal-3 in *P. brasiliensis* infection, indicating that increased Gal-3 production during *P. brasiliensis* infection may affect fungal growth and EV stability, thus promoting beneficial effects that could influence the course of PCM. The finding that Gal-3 has effects against *P. brasiliensis* together with previously reported effects against Cryptococcus neoformans suggests that molecule has a general antifungal role in innate defenses against fungal pathogens.

**IMPORTANCE** Paracoccidioidomycosis (PCM) is the most prevalent systemic mycosis in Latin America. Although the immune mechanisms to control PCM are still not fully understood, several events of the host innate and adaptive immunity are crucial to determine the progress of the infection. Mammalian β-galactoside-binding protein galectin-3 (Gal-3) plays significant roles during microbial infections and has been studied for its immunomodulatory roles, but it can also have direct antimicrobial effects. We asked whether this protein plays a role in Paracoccidioides brasiliensis. We report herein that Gal-3 indeed has direct effects on the fungal pathogen, inhibiting fungal growth and reducing extracellular vesicle stability. Our results suggest a direct role for Gal-3 in *P. brasiliensis* infection, with beneficial effects for the mammalian host.

## Abstract (precis)

Paracoccidioidomycosis (PCM) is the most prevalent systemic mycosis in Latin America. Although the immune mechanisms to control PCM are still not fully understood, several events of the host innate and adaptive immunity are crucial to determine the progress of the infection. Mammalian β-galactoside-binding protein galectin-3 (Gal-3) plays significant roles during microbial infections and has been studied for its immunomodulatory roles, but it can also have direct antimicrobial effects. We asked whether this protein plays a role in Paracoccidioides brasiliensis. We report herein that Gal-3 indeed has direct effects on the fungal pathogen, inhibiting fungal growth and reducing extracellular vesicle stability. Our results suggest a direct role for Gal-3 in *P. brasiliensis* infection, with beneficial effects for the mammalian host.

## INTRODUCTION

Paracoccidioidomycosis (PCM), the most prevalent systemic mycosis in Latin America (1), is caused by the thermodimorphic human pathogens Paracoccidioides brasiliensis and Paracoccidioides lutzii (2). After inhalation of airborne propagules from the fungal mycelium phase, in the lungs, the fungi convert into the infectious form—yeast phase (3–5). The yeast can spread to several organs, causing systemic disease (6). Human defense against PCM depends on a satisfactory cellular immune response and cytokine production (7, 8). Immune mechanisms that prevent cell division and budding of the fungal cells could aid in the control of PCM.

Extracellular vesicles (EVs) are produced by all living cells and actively participate as key regulators of physiopathological mechanisms during fungal infections (9, 10). Fungal EVs carry several virulence factors and other important molecules, contributing to fungal pathogenicity and host immunomodulation (11–16). Since EVs play significant roles in the host-pathogen relationship, vesicular stability is important to ensure suitable delivery of their cargo into host cells (15, 17).

Bacterial and eukaryotic pathogens present surface glycans that may be recognized by host carbohydrate-binding proteins. These interactions commonly affect the microorganism pathogenesis, the host immune response, or the success of intracellular parasitism (18–21). Recently, we reported that galectin-3 (Gal-3), a β-galactoside-binding animal lectin, plays significant roles in cryptococcal infection (15). Gal-3 interferes with Cryptococcus neoformans infection, inhibiting C. neoformans growth and promoting vesicle disruption (15). Also, Gal-3 has been reported to influence the outcome of other mycoses, such as Candida albicans (22) and Histoplasma capsulatum (23). In Paracoccidioides brasiliensis, Gal-3 was reported to play an immunomodulatory role in the host response (24). Since Gal-3 can influence host response against PCM, as well as several other microbial infections, and regulates different functions in the physiopathology of infections, we explored whether Gal-3 influences *P. brasiliensis* growth and vesicle stability.

In this work, we assessed Gal-3 levels in humans and mice with PCM. Also, we demonstrated the influence of Gal-3 in *P. brasiliensis* growth and stability of EVs. Our results demonstrate that Gal-3 inhibits growth and budding of *P. brasiliensis* yeast cells and promotes vesicle disruption. Our results suggest that Gal-3 can impact the interaction of *P. brasiliensis* with host cells.

## RESULTS

### Gal-3 is upregulated during PCM.

**FIG 1:**
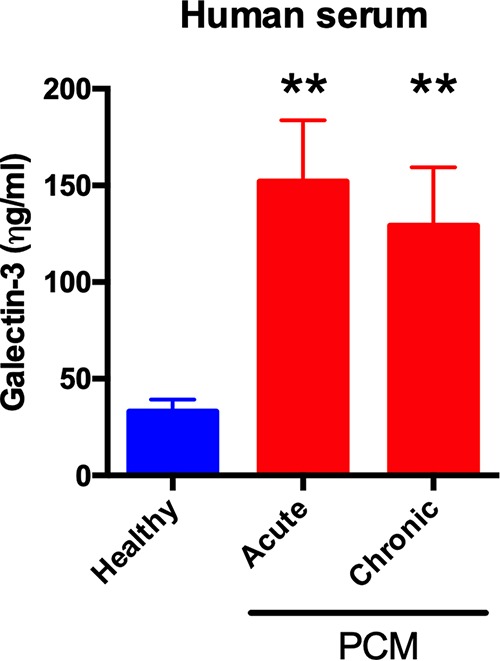
Upregulated Gal-3 levels in humans during *P. brasiliensis* infection. Gal-3 levels in serum from three healthy individuals and patients infected by *P. brasiliensis* (three patients with acute and three patients with chronic form) were assessed by ELISA. Gal-3 levels were higher in acute and chronic form patients infected with *P. brasiliensis* than in healthy individuals. Bars represent the means ± standard deviations (SDs) of Gal-3 levels obtained from triplicate samples. **, *P* < 0.005, two-tailed Student’s *t* test.

Since increased Gal-3 expression was previously described during human and experimental inflammatory diseases (25, 26), and recently in C. neoformans infection (15), we measured Gal-3 levels in serum samples from individuals suffering from PCM, either the acute or chronic PCM form. Compared to healthy individuals, the acute and chronic form patients showed higher Gal-3 serum levels, as shown previously for other infections (Fig. 1). There was no significant difference (*P* value of 0.4204, unpaired *t* test) between the Gal-3 levels in sera of acute and chronic patients with PCM.

**FIG 2:**
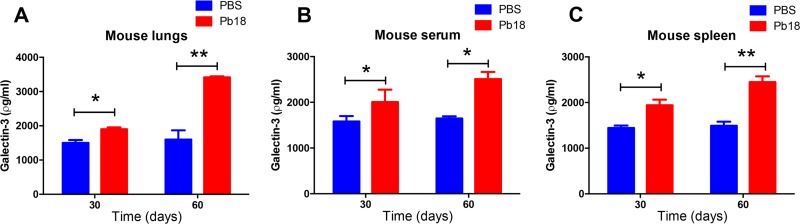
Upregulated Gal-3 levels in mice during experimental *P. brasiliensis* infection. C57BL/6 mice were intratracheally infected with Pb18 strain yeast cells or PBS, and Gal-3 levels were assessed in tissues and serum during the course of *P. brasiliensis* infection. On days 30 and 60 after infection, samples collected of lung (A), serum (B), and spleen (C) tissues were homogenized and Gal-3 quantified by ELISA. Bars represent the means ± SDs of Gal-3 levels obtained from triplicate measurements for each animal, with five animals per group. *, *P* < 0.05; **, *P* < 0.005, two-tailed Student’s *t* test.

Subsequently, we measured Gal-3 levels in tissues and serum of C57BL/6 mice on days 30 and 60 postinfection with *P. brasiliensis* (Fig. 2). In comparison with control animals (phosphate-buffered saline [PBS]), infected mice had higher Gal-3 levels in all examined tissues (lungs and spleen) (Fig. 2A and C, respectively) and serum samples (Fig. 2B). As previously reported for C. neoformans infection, there was a correlation between *P. brasiliensis* infection and increased levels of serum Gal-3, which might reflect the inflammatory conditions caused by these infectious diseases.

### Gal-3 inhibits *P. brasiliensis* growth.

**FIG 3:**
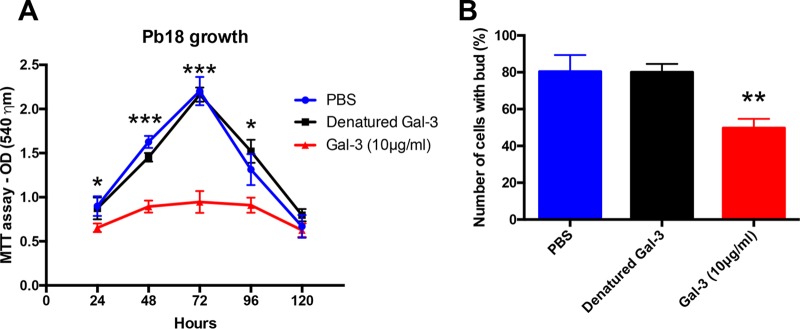
Gal-3 inhibits the growth and budding of *P. brasiliensis* yeast cells. (A) MTT assay of *P. brasiliensis* Pb18 strain cultivated in YPD medium for 120 h at 37°C with 10 μg/ml of Gal-3 or denatured 10 μg/ml Gal-3. (B) Numbers of cells with buds in YPD medium in the absence or presence of denatured Gal-3 or 10 μg/ml Gal-3 for 72 h at 37°C. Buds were counted via light microscopy and quantified using a Neubauer chamber hemocytometer. Data are representative of three experiments, showing means ± SDs for each data point. *, *P* < 0.05; **, *P* < 0.005; ***, *P* < 0.0005, two-tailed Student's *t* test.

Since *P. brasiliensis* cell division and budding are crucial to successful PCM, and Gal-3 inhibits C. neoformans growth (15), we evaluated whether Gal-3 could affect the growth and budding of *P. brasiliensis*. *P. brasiliensis* growth in culture, measured by 3-(4,5-dimethyl-2-thiazolyl)-2,5-diphenyl-2H-tetrazolium bromide (MTT) assay, was compared between Gal-3-treated, PBS-, and denatured Gal-3-treated yeasts. Gal-3 inhibited *P. brasiliensis* growth by approximately 50% after 72 h compared with that of the controls (denatured Gal-3 treated or PBS) (Fig. 3). To verify whether Gal-3 treatment of fungal yeast induces yeast death, we performed viability assays using fluorescein diacetate/ethidium bromide staining. Gal-3-treated and control cultures contained similar proportions of viable cells up until 72 h after Gal-3 treatment, and all cultures were >80% viable (data not shown). To further characterize Gal-3 effects in the growth of *P. brasiliensis*, we evaluated the average number of cells with buds in the yeast culture in the presence or absence of Gal-3 for 72 h, as well as in the presence of denatured Gal-3. We counted the budded or unbudded yeast cells via direct observation in a Neubauer chamber (Fig. 3B). The average number of budding cells was 80% in both untreated and denatured Gal-3-treated cells. On the other hand, the average decreased to 49% in Gal-3-treated cells.

**FIG 4:**
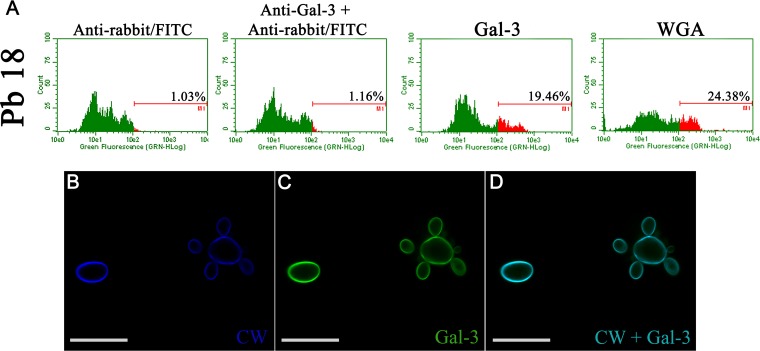
Gal-3 binds to *P. brasiliensis* cell wall. (A) *P. brasiliensis* Pb18 strain cultivated in YPD medium for 72 h at 37°C was resuspended in PBS and incubated sequentially with 40 μg/ml of Gal-3, an anti-Gal-3 antibody, and finally anti-rabbit IgG-FITC antibody. Binding was measured by flow cytometry; numbers inside histograms represent the percentages of positive cells recognized by Gal-3. WGA lectin-FITC (30 μg/ml) was the control for binding with cell wall (CW). (B) *P. brasiliensis* Pb18 strain cultured at 37°C for 72 h and incubated with Gal-3 was stained for observation of cell wall with calcofluor white (blue) (B) and Gal-3 with anti-Gal-3 antibody (green) (C). (D) Merged image (cell wall and Gal-3). The images represent a single section from a Z series stack. Scale bars, 10 μm. Data are representative of three experiments and a representative image is shown.

Flow cytometry assessment of the Gal-3 binding to the *P. brasiliensis* Pb18 strain showed that Gal-3 bound to *P. brasiliensis* cells (Fig. 4A). Confocal microscopy demonstrated that Gal-3 colocalized with calcofluor white, a cell wall dye (Fig. 4B to D). Calcofluor white was used as a positive control as it binds to the cell wall (CW). These results suggest that the recognition of the fungal cell wall by Gal-3, through an unknown sugar moiety, may explain its inhibitory effect on *P. brasiliensis in vitro* growth.

### Gal-3 disrupted *P. brasiliensis* EVs.

**FIG 5:**
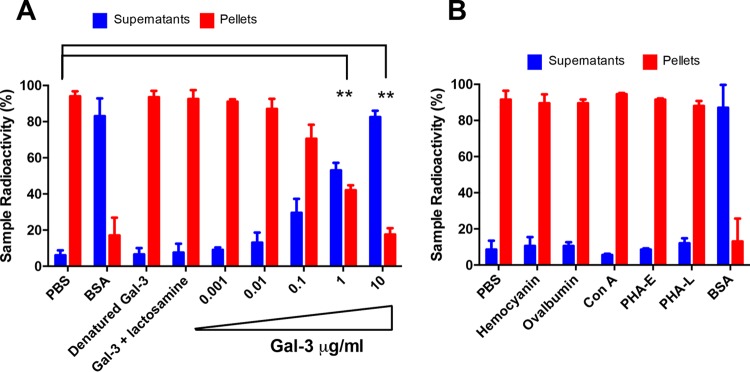
Gal-3 disrupts *P. brasiliensis* extracellular vesicles. (A) Purified radiolabeled vesicles 72 h after [1-^14^C]palmitic acid addition were resuspended in PBS, BSA, denatured Gal-3, Gal-3 preincubated with lactosamine, or Gal-3 (0.001 to 10 μg/ml), and the release of radioactivity from vesicle pellets was assayed. Supernatant and pellet radioactivity was assessed and normalized to 100% radioactivity for each individual sample. (B) Hemocyanin, ovalbumin, concanavalin A (ConA), phytohemagglutinin E (PHA-E), phytohemagglutinin L (PHA-L), and bovine serum albumin (BSA) were used as controls. Bars represent the means ± SDs from triplicate samples from three representative experiment. **, *P* < 0.005, two-tailed Student’s *t* test.

Exposure of EVs produced by C. neoformans to Gal-3, macrophages, or bovine serum albumin causes vesicular disruption (15, 17). We asked whether Gal-3 would affect the stability of EVs produced by *P. brasiliensis*. The addition of Gal-3 to radiolabeled EVs promoted vesicular disruption and subsequent radioactive release in a dose-dependent manner (Fig. 5A). Furthermore, we blocked the Gal-3 carbohydrate recognition domain (CRD) by preincubating with *N*-acetyl-lactosamine (lactosamine, Gal-3 glycoligand) and by Gal-3 denaturation (boiling at 100°C for 10 min). Neither denatured nor lactosamine-bound Gal-3 had a lytic effect on *P. brasiliensis* EVs (Fig. 5A), suggesting that an intact three-dimensional (3D) conformation and Gal-3 CRD are important for the Gal-3 lysing activity. Moreover, radioactive assays showed that other lectins were unable to lyse *P. brasiliensis* EVs (Fig. 5B).

### Gal-3 affects macrophages capability to disrupt and internalize EVs.

**FIG 6:**
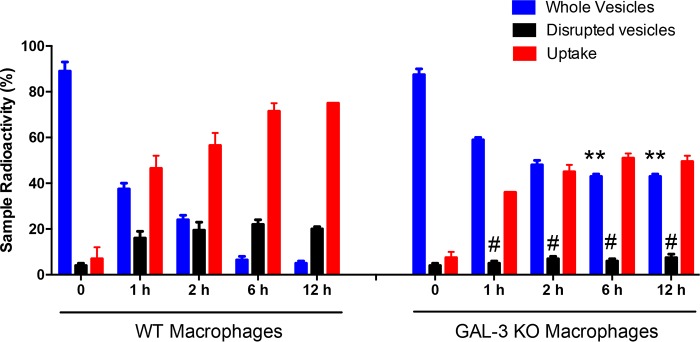
Gal-3 affects disruption and internalization of *P. brasiliensis* EVs by macrophages. Purified radiolabeled EVs from *P. brasiliensis* Pb18 strain yeast cultures were added to cultures of C57BL/6 WT or *Gal-3*^−/−^ macrophages. At 1, 2, 6, or 12 h after EV addition, the radioactivity recovered from the macrophages (adhered cells, uptake), whole vesicles (pellet), and disrupted vesicles (supernatant) was quantified. Bars represent the means ± SDs from triplicate samples from 3 representative experiments. #, *P* < 0.05; **, *P* < 0.005, Student’s *t* test.

Given that Gal-3 is expressed and plays myriad roles in macrophage populations (27–29) and the previous observation that macrophages (17) and Gal-3 can disrupt EVs from C. neoformans (15) and *P. brasiliensis* (this study), we evaluated whether Gal-3 binding and EV lysis might be correlated for *P. brasiliensis*. The addition of radiolabeled EVs from *P. brasiliensis* to the macrophages showed that wild-type (WT) peritoneal macrophages were approximately three times more effective than *Gal-3^−/−^* macrophages to disrupt EVs (Fig. 6, black bars). Moreover, we demonstrated that uptake of EVs from *P. brasiliensis* by WT peritoneal macrophages gradually increased, whereas the uptake by *Gal-3^−/−^* peritoneal macrophages increased significantly less (Fig. 6).

## DISCUSSION

Herein, we describe a role for Gal-3 in *P. brasiliensis* infection that parallels recent observations with C. neoformans (15). Galectins are able to regulate positively or negatively host-microbial interactions in respiratory infections according to galectin type, pathogen, and host context (30, 31). Gal-3, a member of the galectin family of β-galactoside-binding proteins, is widely expressed in different cells and plays important roles in biological phenomena, such as inflammation and immunity (30, 32).

Our previous results in experimental models of cryptococcosis showed Gal-3 levels were increased during infection (15). This observation was replicated in human serum, where higher levels of Gal-3 were detected in patients with cryptococcosis than in healthy individuals (15). The low number of patients available for study prevented us from making definitive statements regarding the increase of Gal-3 in human infection, but in a prior study (15), Gal-3 was shown to be increased in infection; therefore, it is very likely that the increase we observed is true in *P. brasiliensis* infection. Moreover, our results show that Gal-3 inhibits the fungal growth and morphogenesis of *P. brasiliensis*, a fungistatic effect of Gal-3 comparable to what was previously verified for C. neoformans (15). In mouse models, deficiency of Gal-3 led to an increased microbe burden and a decreased survival of the animals (15, 22, 24, 33). Furthermore, *Gal-3^−/−^* mice die faster than wild-type mice when infected with *P. brasiliensis* (24). Gal-3 antifungal effects are widely conserved, affecting most (if not all) fungal pathogens and reaffirming Gal-3 as a critical player in antifungal defenses.

Gal-3 promoted disruption of C. neoformans EVs and influenced the uptake of EV content by macrophages (15), and we now replicate these observations for *P. brasiliensis*. The disruption mechanism of EVs remains cryptic. As discussed previously (15), albumin induces vesicle disruption (17), and albumin can bind to fatty acids (34) and sterols (35) and may promote membrane destabilization. However, we cannot propose a similar mechanism for Gal-3-mediated disruption of EVs, particularly since Gal-3 is not known to bind lipids. We showed Gal-3 disrupts EVs in a manner dependent on functional protein structure and the CRD, and we hypothesize the existence of a novel mechanism of EV disruption. Gal-3 binds beta-galactosides, in proteins (36) and on microbe surfaces (specifically the fungal cell wall), and it is likely that a version of this galactoside is displayed on the surface of microbial EVs. Further studies are needed to discern the glycan moiety recognized in fungal EVs (and whether the glycan moiety is associated with proteins or instead to a putative carbohydrate polymer present in EVs) and how this binding triggers collapse of the lipid bilayer to disrupt EVs. One possible explanation of the decrease in disruption of EVs when exposed to Gal-3 knockout (KO) macrophages is that Gal-3 binding facilitates uptake by macrophages (as Gal-3 facilitates ingestion of glycan-covered beads) (37). This could work in tandem with direct EV-lytic mechanisms by Gal-3. Future work is needed to discern the relative contributions of each of these possible mechanisms. In any case, since EVs from *P. brasiliensis* are reported to modulate macrophage response (13), this may constitute an important mechanism of immune defense: Gal-3 lysis of EVs would prevent EVs’ delivery of a concentrated cargo of molecules relevant to virulence at the host cell surface and instead result in diluted release of fungal components into the extracellular milieu and heightened degradation by host extracellular enzymes.

The Gal-3 serum concentrations were measured by using a commercial kit, which showed that the Gal-3 concentrations in the serum samples of patients with paracoccidioidomycosis ranged from 0.125 to 0.150 μg/ml (Fig. 1). In the dose-response curve measuring if recombinant Gal-3 concentrations were capable of disrupting of *P. brasiliensis* vesicles (Fig. 5), we assayed 1 μg/ml of Gal-3 and verified this concentration was sufficient to disrupt vesicles, compared to the degree of lysis in the PBS control, which is attributable to spontaneous disruption. We had used this 10 μg/ml concentration previously in tests for vesicle stability for EVs produced for C. neoformans (15), and we had observed almost complete disruption of EVs at 10 μg/ml of Gal-3. We note that the Gal-3 concentration needed to disrupt EVs or to inhibit fungal growth is much higher than the concentrations of Gal-3 measured in human and mouse tissues (>60-fold and 2,000-fold higher than concentrations of human serum and mouse tissues, respectively); therefore, the magnitude of Gal-3-mediated direct antimicrobial effects occurring *in vivo* is still unclear. Despite the lower concentration of Gal-3 measured in mouse and human serum, local concentrations in infected tissues may be much higher than those measured in serum (38) or recoverable by our extraction techniques. Consequently, a concentration of 1 to 10 μg/ml may be biologically relevant. Since higher Gal-3 levels are required *in vitro* to promote vesicle lysis and inhibit fungal growth than the detected Gal-3 levels in tissues, future studies will be aimed to better understand the mechanism of antimicrobial effects *in vivo*.

We conclude that Gal-3 is beneficial for the mammalian host during *P. brasiliensis* infection by contributing to host defense. As previously reported for C. neoformans (15), the antimicrobial mechanism of Gal-3 is due to microbial vesicle lysis coupled with inhibition of fungal growth and morphogenesis. In addition to direct antimicrobial effects, Gal-3 plays immunomodulatory roles (33, 39–41) that may synergize with the antimicrobial effects. Multiple inhibitors of Gal-3 have been designed (42) and are being tested for antitumorigenic properties. However, our data reveal that these therapies may predispose patients for fungal infections, and as is the case for many immunotherapies, it is important to closely monitor fungal infections in these patients. In the case of fungal infections, it may be desirable to design a Gal-3 mimetic that, through inhibition of growth and interference with EV release, would act as a potent antifungal therapy.

## MATERIALS AND METHODS

### Ethics statement.

All animal use complied with the standards described in Ethical Principles Guide in Animal Research adopted by the Brazilian College of Animal Experimentation. The protocols were approved by the Committee of Ethics in Animal Research of the Ribeirao Preto Medical School at the University of Sao Paulo (protocol 20/2013-1). Informed written consent from all participants was obtained. The studies involving patients were approved by the Research Ethics Committee of the University Hospital, Ribeirao Preto Medical School, at the University of Sao Paulo (protocol HCRP 13.982/2005).

### Mice and *P. brasiliensis* strain.

We used male C57BL/6 (wild type [WT]; Jax 000664) and galectin-3-deficient (*Gal-3^−/−^*) mice at 6 to 8 weeks of age. Knockout mice were kindly donated by F. T. Liu (University of California, Davis, CA). *Gal-3^−/−^* mice were previously generated as described and crossbred to the C57BL/6 mouse background for nine generations (43). The animals were housed in the animal facility of the Ribeirao Preto Medical School, University of São Paulo, under optimized hygienic conditions. All *P. brasiliensis* experiments were performed with the Pb18 isolate. Fungal cultures were grown in YPD medium (2% peptone, 1% yeast extract, and 2% glucose) in the yeast phase at 36°C. To ensure yeast virulence, serial passages in BALB/c mice were performed before the isolate Pb18 was used in experiments.

### Sera and patients.

Blood samples were obtained from patients being seen in the University Hospital, Ribeirao Preto Medical School, at the University of Sao Paulo. A total of 6 patients with diagnosed paracoccidioidomycosis were included in this study: 3 patients with acute and 3 patients with chronic form. Serum was obtained and stored at −80°C. Samples were also obtained from 3 healthy blood donors with a median age of 30 years (range, 25 to 35 years).

### Gal-3 levels.

Gal-3 levels in the lung, serum, and spleen were quantified in the organ homogenates of *P. brasiliensis*-infected mice. The homogenate samples (whole organ in 1 ml of PBS) of control mice or from animals infected with *P. brasiliensis*, as well as the serum samples from infected animals or from patients with diagnosed paracoccidioidomycosis, were stored at −80°C until assayed. All samples were thawed only once prior to use. Gal-3 levels were measured using commercially available enzyme-linked immunosorbent assay (ELISA) kits (Sigma-Aldrich, St. Louis, MO, USA) according to the manufacturer’s instructions.

### Cell viability and growth in the presence of Gal-3.

Fungal viability was determined using fluorescein diacetate/ethidium bromide staining, as previously described (44). Only the cultures that were greater than 85% viable were used.

To verify Gal-3 effect on the cells, we performed growth curves in YPD liquid medium containing different concentrations of Gal-3 (Gal-3 human recombinant, expressed in Escherichia coli; Sigma-Aldrich) in a 96-well plate (Costar, NY, USA). The Gal-3 effect on cell proliferation was determined using an MTT assay (45), as follows: *P. brasiliensis* yeast cells were suspended in YPD medium at a density of 10^6^ cells/ml and treated with Gal-3, denatured Gal-3, or PBS as a control after incubating at 37°C in an orbital shaker (150 rpm) for 120 h. To verify the Gal-3 effect on yeast budding, we assessed the average number of Pb18 strain cells with buds found in yeast cultures after 72 h of Gal-3 treatment versus cells treated with denatured Gal-3 and PBS. Counting was carried out in a Neubauer chamber by optical microscopy, considering the yeasts that presented at least one or more buds as budding cells.

### Gal-3 binding assay and confocal microscopy.

The Gal-3 binding assay and confocal microscopy were performed as previously described for C. neoformans (15) using *P. brasiliensis* yeast cells. Gal-3 binding assay was performed with yeast cells incubated with PBS containing 10% fetal bovine serum for 20 min at 4°C to block nonspecific binding of antibodies. Next, 1 ml of the suspension containing 10^6^ cells was incubated with either Gal-3 (40 µg/ml) or denatured Gal-3 (40 µg/ml) for 40 min at 4°C. Cells were washed twice with PBS, and anti-Gal-3 antibody (1:50; Sigma-Aldrich) was added; after incubating for 45 min, the cells were washed twice with PBS and incubated with anti-rabbit IgG-fluorescein isothiocyanate (FITC) antibody (1:50; Sigma-Aldrich) for 40 min at 4°C. Gal-3 binding to *P. brasiliensis* cells was analyzed by flow cytometry (Guava easyCyte; Guava Technologies, Millipore, Hayward, CA, USA). The association of anti-rabbit IgG-FITC antibody with Gal-3 and wheat germ agglutinin (WGA) lectin (30 μg/ml), used as negative and positive controls, respectively, was assessed. Confocal microscopy was performed with cells incubated with Gal-3 (40 μg/ml) at 37°C for 1 h, washed three times with PBS, and fixed with PBS-buffered 3.7% formaldehyde at 25°C. The samples were washed three times with PBS and treated with glycine 0.1 M for 15 min and blocked with bovine serum albumin (BSA; 1% in PBS) for 1 h at 25°C. Then, the cells were incubated with a rabbit anti-Gal-3 antibody (Sigma-Aldrich) overnight at 4°C. The samples were washed five times with PBS and incubated for 1 h with FITC-labeled donkey anti-rabbit IgG from Jackson ImmunoResearch Laboratories. For cell wall staining, samples were incubated with calcofluor white (50 μg/ml; Sigma-Aldrich) in PBS for 20 min. After five washes with PBS, cells were placed on slides and coverslips were mounted with Fluoromount-G (Electron Microscopy Sciences). The samples were examined with an LSM780 system Axio Observer, with a 63× oil immersion objective (Carl Zeiss, Jena, Germany). The images were analyzed offline using ImageJ software (http://rsb.info.nih.gov/ij/). Secondary antibody alone was used as the control. All controls were negative.

### Analysis of the stability of extracellular vesicles.

EVs were isolated as previously described (46). Vesicle quantification was measured by nanoparticle-tracking analysis (NTA) using a NanoSight NS300 (Malvern Instruments, Malvern, UK) equipped with fast video capture and particle-tracking software, as previously described (16). Purified vesicles from *P. brasiliensis* were diluted in PBS, and each sample was then injected into a NanoSight sample cubicle. The measurements were obtained in triplicates and analyzed using NanoSight software (version 3.2.16). EV stability was evaluated according to protocols previously described (15). EVs were incubated with Gal-3 (Gal-3 human recombinant, expressed in E. coli; Sigma-Aldrich) at different final concentrations (0 to 10 µg/ml), and the concentrations of all control lectins were normalized according to carbohydrate binding sites. EV stability was examined by radioactive assay through cultivation of *P. brasiliensis* in the presence of [1-^14^C]palmitic acid, as previously described for C. neoformans (15, 17). The suspension of radiolabeled EVs was incubated with Gal-3 at 37°C for different times and concentrations, and the suspension was ultracentrifuged at 100,000 × *g* for 1 h at 4°C. Supernatants and pellets were saved for scintillation counting.

### Vesicle disruption and uptake by macrophages.

To assess the vesicle stability and vesicle uptake by macrophages from WT and *Gal*-*3*^−/−^ mice, we used a protocol previously described (15). Peritoneal macrophages were obtained from C57BL/6 WT or *Gal-3*^−/−^ mice and grown in 90% Dulbecco’s modified Eagle’s (DME) medium (Invitrogen) and 10% NCTC medium (Invitrogen) supplemented with fetal bovine serum, nonessential amino acids (Invitrogen), and penicillin (Invitrogen). Forty-eight-well tissue culture plates were seeded with thioglycolate-elicited peritoneal macrophages (4 × 10^5^ cells/well). EVs were obtained from *P. brasiliensis* cultures that were pulsed with [1-^14^C]palmitic acid 72 h before EV harvesting and added to macrophage cultures as previously described (15). Then, the culture supernatant was harvested from macrophage-EV coculture and centrifuged (100,000 × *g* for 1 h at 4°C) to separate soluble and EV-containing fractions of the macrophage cell culture supernatant. Thus, we obtained three different samples: the adhered cells (containing EVs due to uptake), supernatants (containing components of disrupted EVs), and pellets (containing intact EVs). These samples were saved for scintillation counting. The radioactivity distribution in the three fractions was expressed as percentage of the total radioactivity.

### Statistical analysis.

Data are either the means or representative results from at least 3 independent experiments, each performed in triplicates. All statistical analyses and comparisons were performed using GraphPad Prism software version 6.0 (GraphPad Software, San Diego, CA, USA). A *P* value of <0.05 was considered statistically significant.
